# Supplementary material for: Comparison of Anti-Obesity Effects of Ginger Extract Alone and Mixed with Long Pepper Extract
Source: Biomedicines. 2025 Aug 26;13(9):2077. doi: 10.3390/biomedicines13092077 (PMC12467498; doi:10.3390/biomedicines13092077)
Supplement: Supplementary file 1 [file biomedicines-13-02077-s001.zip › biomedicines-3813691-supplementary.pdf]

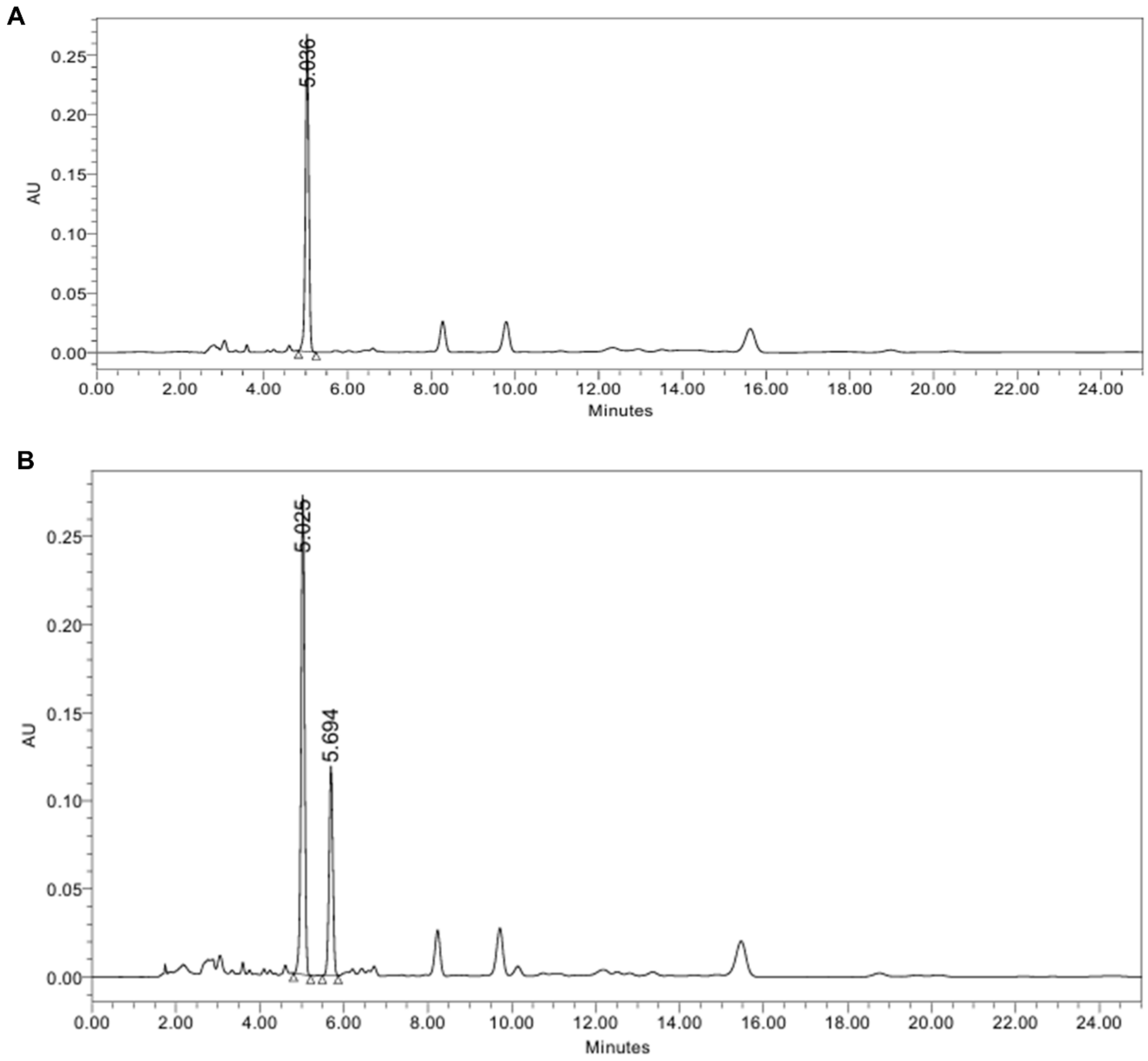

Figure S1. Representative high-performance liquid chromatography (HPLC) chromatograms: (A) GE, showing 6-gingerol at 5.036 min; and (B) a GE and LPE mixture, showing a distinct peak for piperine at 5.694 min.
